# Immunological evaluation of patients with orthopedic infections: taking the Cierny–Mader classification to the next level

**DOI:** 10.5194/jbji-6-433-2021

**Published:** 2021-12-01

**Authors:** Janet D. Conway, Vache Hambardzumyan, Nirav G. Patel, Shawn D. Giacobbe, Martin G. Gesheff

**Affiliations:** 1 International Center for Limb Lengthening, Rubin Institute for Advanced Orthopedics, Sinai Hospital of Baltimore, Baltimore, Maryland, USA; 2 Armenian-American Wellness Center, Yerevan, Armenia

## Abstract

**Introduction**: Cierny–Mader osteomyelitis classification is used to label A, B, or C hosts based on comorbidities. This study's purpose was to define the “true” host status of patients with orthopedic infection using serologic markers to quantify the competence of their immune system while actively infected. **Methods**: Retrospective chart review identified patients at a single-surgeon practice who were diagnosed with orthopedic infection between September 2013 and March 2020 and had immunological laboratory results. All patients were A or B hosts who underwent surgery to eradicate infection. Medical history, physical examination, and Cierny–Mader classification were recorded. Laboratory results included complement total, C3, C4, immunoglobulin G (IgG), immunoglobulin M (IgM), immunoglobulin A (IgA), immunoglobulin E (IgE), rheumatoid factor, and antineutrophil cytoplasmic antibodies (ANCA) panel. Clinically significant results were defined as flagged abnormal. Normal complement levels and normal IgG levels were considered abnormal when infection was present. **Results**: Of 105 patients, 99 (94 %) had documented lab abnormalities. Clinically significant abnormalities were found in 33 of 34 (97 %) type-A hosts and 66 of 71 (93 %) type-B hosts. Eleven of 105 (10.5 %) patients were formally diagnosed with primary immunodeficiency by a hematologist. IgG deficiency, of either low or normal value, in the face of infection comprised 91 % (30 of 34) type-A hosts and 86 % (56 of 71) type-B hosts. Six (5.7 %) patients received IgG replacement therapy. Twenty-eight patients had abnormal total complement levels (low or normal): 7.4 % (2 of 34) A hosts and 40 % (26 of 71) B hosts ( p=0.002 ). B hosts had statistically significantly lower complement levels and significantly more no-growth cultures ( p<0.03 ). Thirteen of 14 patients with recurrent infections had low or normal IgG levels. IgM was significantly lower between reinfected patients and those without reinfection ( p=0.0005 ). **Conclusions**: Adding immunologic evaluation to the Cierny–Mader classification more accurately determines patients' true host status and better quantifies risk and outcome with respect to orthopedic infection. Immunologically deficient A hosts should be quantified as B hosts. IgG deficiencies may be addressed when deemed appropriate by the consulting hematologist/immunologist. Patients with recurrent infections had significantly lower IgM levels than their nonrecurrent infection counterparts.

## 1 Introduction

Orthopedic infections are a catastrophic consequence not only for the patient, but also for the healthcare system. Since the introduction of the Cierny–Mader osteomyelitis classification, hosts have been labeled A, B, or C based on comorbidities from their history and physical evaluation (Cierny et al., 2003). In most cases, susceptibility factors for infection are readily identifiable; however, for some type-A hosts, the contributing factors may remain obscure. When all other factors are absent and the host is presumably “normal” (i.e., A host), a deficient immune system predisposes a patient to an increased risk of infection. A hosts may not have had any reason prior to their orthopedic surgery to suspect that their immune system is deficient. The perpetual question is why patients become infected. In this era of accountability in healthcare, it is essential to evaluate and optimize all patients to minimize orthopedic infection risk. Secondary causes of immunodeficiencies are well documented in the literature, including conditions such as viral infection, chemotherapy, nephrotic syndrome, protein-losing enteropathy, hematological malignancy, and immunosuppressive or anticonvulsant medications (Chinen and Shearer, 2010). However, primary immunodeficiency disorders (PIDs) result from immune system defects and play an important role in a patient's host status (Driessen and van der Burg, 2011; McCusker et al., 2018). In pediatric patients with osteomyelitis and septic arthritis, Beard et al. (1990) found that “impaired antibody production may be a predisposing factor” for septic arthritis and osteomyelitis development. Bloom et al. (2008) analyzed osteoarticular infectious complications in patients with already diagnosed PID and reported that septic arthritis is a significant complication of PID. Serum level of immunoglobulin G (IgG), immunoglobulin A (IgA), immunoglobulin E (IgE), and immunoglobulin M (IgM) as well as autoimmune markers such as anti-nuclear antibodies (ANAs), rheumatoid factor (RF), and complements C3, C4, and CD27 + provide clues to subtle primary immunodeficiency in otherwise healthy A hosts (Gonzalez-Quintela et al., 2008; Ekdahl et al., 2018; Filion et at., 2019). Serological evaluation preoperatively can elucidate hidden immunologic risk factors that predispose an otherwise “normal” population to an increased incidence of infection. Many variants of PID exist, with symptoms ranging from mild to severe. Many patients are not even diagnosed until they are in their third decade of life (Cooper et al., 2003), coincidentally coinciding with many common orthopedic procedures such as rotator cuff repairs, knee arthroscopy for torn menisci, and other “weekend-warrior”-type overuse injuries.

Host optimization in the setting of orthopedic infection is necessary and may require consideration beyond the obvious risk factors, especially in patients with seemingly normal general health who are suffering from infectious complications. Preoperative screening could allow immunological abnormalities in this “silent” population to be identified and treated before surgery to prevent postoperative orthopedic infections.

The purpose of this study was to describe the immunological abnormalities observed in our infected orthopedic patient population, use this information to define their true host status, determine which serologic variables had the largest correlation with poor outcomes, and ultimately optimize their orthopedic surgical outcomes. We know of many possible factors that increase patients' risk; however, no one has defined the role of the patients' own immune system in contributing to this problem. This paper begins to shed light on these contributing factors to infection risk, especially when all other factors are accounted for and managed. This study reports a captured population of surgically treated, infected orthopedic patients stratified into host status in combination with an evaluation of their immune system based upon serologic immune markers.

## 2 Methods

The LifeBridge Health Institutional Review Board (IRB no. 2079) reviewed this study and provided a determination of exempt status for this retrospective review. The investigation was performed in accordance with the principles of the Declaration of Helsinki. Informed consent was not required. Starting in 2013, all patients presenting with orthopedic infection were evaluated for medical comorbidities and serologic abnormalities as part of the standard preoperative workup in a single-surgeon practice. A retrospective chart review was conducted to identify a cohort of patients diagnosed with orthopedic infection at our institution with available immunological laboratory results between September 2013 and March 2020. All patients with suspected orthopedic infection had undergone lab work, as well as patients with aseptic joint loosening (in the event it was the result of indolent infection). All patients underwent surgery to eradicate the infection, therefore making all the patients either A or B hosts. C hosts were nonsurgical candidates and were not included for preoperative serological evaluation.

All A and B hosts were tested preoperatively. Medical history and physical examination were recorded to stratify patient host status according to the Cierny–Mader classification system. The local tissue environment was not included in the host classification because the local tissue conditions were not relevant to the overall immunocompetency of the patient. Laboratory results recorded included complement total, C3, C4, IgG, IgM, IgA, IgE, rheumatoid factor, and antineutrophil cytoplasmic antibodies (ANCA) panel. Patients with abnormalities were referred to hematology/immunology. In selected IgG deficiency cases, patients received preoperative immunoglobulin replacement therapy from the hematologist/immunologist. In preoperatively diagnosed cases, surgical intervention was postponed to replenish IgG. If labs were obtained during the hospital stay, IgG was administered during the surgical hospital stay. For patients who had abnormalities that were not readily correctable, measures were taken to boost nutrition status. For those receiving replenishment of IgG deficiencies, monthly IgG levels were monitored. As part of our patient treatment protocol, if these patients needed additional surgical intervention after completion of orthopedic infection treatment, they were advised to tell their surgeon about their previous deficiency and obtain a preoperative immunology panel prior to the next surgery (often unrelated to the previous surgical site) in an effort to maximize infection prevention. Post treatment immunologic markers tested are similar to the preoperative panel and include total complement level, C3, C4, IgG, IgA,IgM, IgE, ANCA, and RF. Repeat testing was instituted when the infection was cleared for a minimum of 6 months with normal serologic C-reactive protein (CRP) and erythrocyte sedimentation rate (ESR). Patients who were on oral prophylactic suppressive antibiotics were not tested until 8 weeks after the completion of the oral course. Treatment failure was defined as the presence of recurrent infection after the index procedure in this cohort.

Clinically significant results were defined as flagged abnormal (based on standard lab reference ranges because patients were not standardized to one lab) low or normal IgG, low IgM, high IgE, low or high IgA, low total complement, positive ANCA (ANCA atypical, ANCA myeloperoxidase (MPO), C-ANCA, P-ANCA), and positive rheumatoid factor (McCusker et al., 2018; Bonilla et al., 2016). Normal complement levels and normal IgG levels were considered abnormal in the face of infection. Abnormality was considered based upon on the reference laboratory utilized for each patient, and this report specifically describes either high, normal, or low rather than actual quantitative results. Please note that not all patients had the full immunological panel: we report on a full cohort of patients and all available laboratory results located in the medical record.

All data were recorded using Excel (Microsoft Office, 2011, Redmond, Washington). All statistical analyses were performed using MedCalc Statistical Software version 19.0.7 (MedCalc Software bvba, 2019). Descriptive statistics were calculated; comparisons of proportions were compared with a chi-squared test or Fisher's exact test for comparison of less than five cases per cell.

## 3 Results

After a clinic visit for their orthopedic infection, 178 patients were prescribed immunological lab workup. Thirty-nine patients did not complete the lab workup and were not included in the study. Of the 144 remaining patients, 105 had documented infection by the Musculoskeletal Infection Society criteria (Parvizi et al., 2018) and underwent surgery for orthopedic infection (Table 1). Surgical debridement was performed using a separate operative setup. All bone was resected to clean and bleeding bone with a high-speed well-irrigated burr. Intramedullary canals were sequentially reamed to clean bone. Necrotic tissue was sharply resected with a scalpel, Bovie knife, and hydroscalpel. Six liters of irrigation was then used with a 10 min dilute betadine soak while all gowns, gloves, and drapes were replaced. A new clean setup was used for the reconstruction.

**Table 1:** Patient demographics.

	Total group	Type-A host	Type-B host	P value
Host status N or % ( N of total)	105	32 % (34)	68 % (71)	–
Age mean ± SD	57.8±13.9	54.1±15.2	59.6±13.0	0.0581
Male gender % ( N )	52 % (55)	56 % (19)	51 % (36)	0.6228
Infection location				
Knee	49.5 % (52)	44.1 % (15)	52.1 % (37)	0.1620
Hip	14.3 % (15)	23.5 % (8)	9.9 % (7)	
Tibia	8.6 % (9)	8.9 % (3)	8.5 % (6)	
Ankle	7.6 % (8)	0 % (0)	11.3 % (8)	
Femur/thigh	6.7 % (7)	11.8 % (4)	4.2 % (3)	
Shoulder	3.8 % (4)	5.9 % (2)	2.8 % (2)	
Foot	3.8 % (4)	2.9 % (1)	4.2 % (3)	
Elbow	2.9 % (3)	0 % (0)	4.2 % (3)	
Lower leg	1.0 % (1)	2.9 % (1)	0 % (0)	
Fibula	1.0 % (1)	0 % (0)	1.4 % (1)	
Humerus	1.0 % (1)	0 % (0)	1.4 % (1)	
Hematology/immunology diagnosis % ( N of total) consulted	10.4 % (11)	8.8 % (3)	11.2 % (8)	–
Hypogammaglobulinemia	72.7 % (8)	66.7 % (2)	75 % (6)	1.0000
Anemia	45.5 % (5)	33.3 % (1)	50 % (4)	1.0000
IgM deficiency	9.1 % (1)	0 % (0)	12.5 % (1)	1.0000
Leukocytosis	9.1 % (1)	0 % (0)	12.5 % (1)	1.0000
IgA deficiency	9.1 % (1)	33.3 % (1)	0 % (0)	0.3333
IgG replacement post consult % ( N )	5.7 % (6)	2.9 % (1)	7.0 % (5)	1.0000

IgA: immunoglobulin A; IgG: immunoglobulin G; IgM: immunoglobulin M.

Culture protocol is three to five intraoperative tissue samples as well as joint fluid. For culture-negative infections, after 5 d the lab was requested to hold the plates for 2 weeks total. Six weeks of culture-specific antibiotics were administered either orally or intravenously. For culture-negative infections, any previous culture information was obtained and antibiotics were given based upon that information. When no information was available, broad-spectrum antibiotics were administered, usually vancomycin in combination with a third-generation cephalosporin based upon recommendations from the infectious disease consultant.

There were 55 males and 50 females included in this cohort, with a mean clinical follow-up of 404 d ( ±398 ). Thirty-four (32 %) patients were clinically classified as A hosts, and 71 (68 %) patients were clinically classified as B hosts. The mean age of the A hosts was 54.1 years ( ±15.2 ); the mean age of the B hosts was 59.6 years ( ±13.0 ). There was no statistically significant difference between age or gender between the A or B hosts, with p values of 0.058 and 0.6208, respectively. The most common procedures for infection were performed in the region of the knee and hip (Table 1). The most common organisms were culture-negative, followed by coagulase-negative staphylococci and *Staphylococcus aureus* (Table 2). Culture-negative infections often can occur as a result of low-virulence organisms and mycoplasma. These types of infections often occur in hosts with weakened immune systems such as those B hosts in this study. Mycoplasma was not routinely tested in these cultures. There was no significant difference between A and B hosts with respect to organisms and index procedure.

**Table 2:** Organism growth with incidence comparison between host status.

	Total group	Type-A host	Type-B host	P value
Index procedure organism				
No growth	39 %	24 %	46 %	0.0313
Coagulase-negative staphylococci	14 %	12 %	15 %	0.7730
*Staphylococcus aureus*	12 %	18 %	10 %	0.2498
Corynebacterium	8 %	9 %	7 %	0.7154
MRSA	6 %	12 %	3 %	0.1783
*Enterococcus* (*faecalis*, group D, vancomycin resistant)	6 %	3 %	7 %	0.6629
*Pseudomonas aeruginosa*	4 %	6 %	3 %	0.5975
*Candida* (*albicans, magnoliae, parapsilosis*)	4 %	3 %	4 %	1.0000
*Enterobacter* (*aerogenes, cloacae*)	4 %	3 %	4 %	1.0000
*Streptococcus* (*agalactiae B, pyogenes A, viridans*)	6 %	6 %	6 %	1.0000
*Bacteroides* (unspecified, *fragilis*)	3 %	6 %	1 %	0.2572
Other (*E. coli*, *Klebsiella pneumonaie*, *Proteus mirabilis*, *Bacillus* (not anthracis), *Cutibacterium* (propionibacterium) *acnes*, *Fusobacterium nucleatum*, *Haemophilus parainfluenzae*, *Peptostreptococcus*, *Prevotella bivia*, *Serratia marcescens*, *Staphylococcus lugdunensis*)	12 %	21 %	9 %	0.0874
Multiple organism incidence	13 %	18 %	11 %	0.3248
Reinfection organism				
No growth	50 %	43 %	58 %	1.0000
*Escherichia coli*	14 %	14 %	14 %	1.0000
*Achromobacter xylosoxidans*	7 %	14 %	0 %	1.0000
*Bacteroides* species	7 %	14 %	0 %	1.0000
*Corynebacterium* species	7 %	14 %	0 %	1.0000
*Enterococcus faecalis*	7 %	14 %	0 %	1.0000
MRSA	7 %	14 %	0 %	1.0000
Coagulase-negative staphylococci	7 %	0 %	14 %	1.0000
*Staphylococcus aureus*	7 %	0 %	14 %	1.0000
*Staphylococcus epidermidis*	7 %	14 %	0 %	1.0000
*Streptococcus sanguinis*	7 %	14 %	0 %	1.0000
Multiple organism incidence	7 %	14 %	0 %	1.0000

MRSA: methicillin-resistant *Staphylococcus aureus*.

Immunology lab data were documented for 105 patients. Lab abnormalities were documented in 99 of 105 (94 %) patients. Clinically significant immune marker abnormalities were observed in 33 of 34 (97 %) type-A hosts and 66 of 71 (93 %) type-B hosts, and there was no statistical significance between these groups when comparing them with respect to any abnormality ( p=0.4138 ) (Table 3). Arrows in the table indicate whether the lab value was abnormally high or low. All patients were referred to a specialist when abnormal immunology values were detected for definitive diagnosis of PID and ultimate management. Eleven patients were formally diagnosed with immunodeficiency by a hematologist.

**Table 3:** Host status comparison of immunological laboratory assessments – clinically significant abnormalities incidence.

	Total group	Type-A host	Type-B host	P value
	N=105	N=34	N=71	
ANCA atypical	4.2 %	4.2 %	4.3 %	1.0
ANCA MPO	0.0 %	0.0 %	0.0 %	–
Complement total ( ↓ or N )	30 %	7.4 %	40 %	0.0021
C-ANCA	0.0 %	0.0 %	0.0 %	–
P-ANCA	1.5 %	0.0 %	2.2 %	0.4945
IgG ( ↓ or N )	88 %	91 %	86 %	0.4995
IgA ( ↓ or ↑ )	20 %	18 %	21 %	0.7247
IgE ( ↑ )	29 %	17 %	35 %	0.0867
IgM ( ↓ or N )	26 %	27 %	25 %	0.8312
RF ( ↑ )	10 %	3.1 %	14 %	0.1088
Any abnormality	94 %	97 %	93 %	0.4138

ANCA: antineutrophil cytoplasmic antibodies; IgA: immunoglobulin A; IgE: immunoglobulin E; IgG: immunoglobulin G; IgM: immunoglobulin M; MPO: myeloperoxidase; RF: rheumatoid factor. Percentages are reported as total patients with the clinically significant abnormal laboratory assessment divided by total number that received the laboratory assessment.

Among abnormal results, IgG deficiency, either low or normal value, in the face of infection comprised 91 % (30 of 34) in type-A hosts and 86 % (56 of 71) in type-B hosts. Six (5.7 %) subjects were prescribed subsequent IgG replacement therapy by a specialist. A total of 86 patients (88 %) had abnormal IgG levels, of which 35 % (30 of 86) were A hosts and 66 % (56 of 86) were B hosts. Infections were successfully eradicated in the six patients receiving IgG therapy.

Of 105 patients, 28 had abnormal total complement levels (low or normal) (A hosts, 7.4 %, 2 of 34; B hosts, 40 %, 26 of 71). This difference in complement levels between host status was statistically significant ( p=0.002 ). Of these 28 patients with low complement, 6 had a diagnosis of hypogammaglobulinemia by a hematologist. These were all quantified as traditional B hosts. Of the 28 patients with low complement levels, 3 had recurrent infections with one organism each (i.e., *E. coli*, *Staphylococcus aureus*, and coagulase-negative staphylococci). This was not statistically significant. Therefore, having just a low complement level did not predispose this group to an increased risk of recurrent infection.

Of the 14 low IgG hosts, 6 cultured no growth, and the rest were infected with various organisms (Table 1). With respect to the 14 patients with recurrent infections, 7 patients had no growth (Table 2). IgM was statistically significantly lower in all patients with recurrent infections when compared to those patients without recurrent infections. This may be a useful tool for predicting the risk of recurrent infection and is currently being tracked in our current population.

Other abnormalities reported included positive ANCA, positive p-ANCA, abnormally low or high IgA, and abnormally high IgE. These abnormalities were not statistically significant between the two host status groups.

Fourteen patients had recurrent or subsequent infections at a second location. Seven of these patients had no growth (Table 2). Thirteen of these 14 patients had low or normal IgG levels, but this percentage of low/normal IgG when compared to the noninfected group was not statistically significantly different ( p=0.48 ). A significant difference was noted in terms of IgM between reinfected patients and those without reinfection ( p=0.0005 ); 58 % of those with reinfection had low IgM (Table 4).

**Table 4:** Reinfection comparison of immunological laboratory assessments – clinically significant abnormalities incidence.

	Known reinfection	No known reinfection	P value
	N=14	N=78	
Host status A	50 %	31 %	0.1641
ANCA atypical	7.7 %	2.1 %	0.3196
ANCA MPO	0.0 %	0.0 %	–
Complement total ( ↓ or N )	43 %	28 %	0.1301
C-ANCA	0.0 %	0.0 %	–
P-ANCA	0.0 %	2.2 %	0.6074
IgG ( ↓ or N )	93 %	86 %	0.4784
IgA ( ↓ or ↑ )	21 %	19 %	0.8642
IgE ( ↑ )	21 %	30 %	0.5433
IgM ( ↓ or N )	58 %	16 %	0.0005
RF ( ↑ )	0.0 %	12.5 %	0.1802
Any abnormality	100 %	94 %	0.3497

ANCA: antineutrophil cytoplasmic antibodies; IgA: immunoglobulin A; IgE: immunoglobulin E; IgG: immunoglobulin G; IgM: immunoglobulin M; MPO: myeloperoxidase; RF: rheumatoid factor. Percentages are reported as total patients with the clinically significant abnormal laboratory assessment divided by total number that received the laboratory assessment.

## 4 Discussion

As healthcare providers who are held accountable for all adverse patient outcomes, these data are relevant and critical. This is the first study to report immunological abnormalities detected in the orthopedic infection population. These immunologic markers are an important adjunct to the Cierny–Mader classification to correctly quantify a patient's “true” host status. Based on the immunological profile, 94 % of the Cierny–Mader A-host patients were not immunocompetent hosts with a normal immune system. If a serologic evaluation is added to the current Cierny–Mader classification, the “immunologically abnormal” A hosts would be correctly categorized as B hosts. This has prognostic implications as B hosts carry higher postoperative complications and a lower percentage of successful outcomes after orthopedic surgery (McPherson et al., 2002). Of the 14 patients with recurrent infections, 7 (50 %) were classified as traditional A hosts. In our population of infected patients, checking serologic immunology markers provided useful information to the surgeon and patient as to some of the potential nonobvious reasons why they may be more susceptible to an orthopedic infection.

Thirteen of the 14 patients with recurrent infections had low IgG. This trend with low IgG and recurrent infection documents how important IgG is in fighting infection. This must be considered when evaluating an orthopedic infection and optimized to reduce reinfection risk.

B hosts had statistically significantly lower levels of complement proteins than the A hosts ( p=0.002 ). The complement system plays a major role in the immune system via a cascade of proteins that fight infection and activate other mechanisms in the immune cascade. Certain bacteria have developed proteins that can interfere with the activation of the complement system, thus weakening the immune response to the bacterial infection. *Staphylococcus aureus* has the ability to dampen complement activation (Sarma and Ward, 2011) and even destroy complement proteins by proteolysis. Also, *Staphylococcus aureus* can express a protein called staphylococcal immunoglobulin-binding protein A, which binds to the Fc portion of the IgG and prevents complement activation and Fc receptor-mediated phagocytosis. It is unclear why the B hosts had more deficient complement levels. Of the 28 patients with low complement, 5 were infected with *Staphylococcus aureus*, 4 were infected with *Staphylococcus epidermidis*, and 11 had no growth on their cultures. The large percentage of no growth on cultures in the low-complement group is disappointing but could be attributed to a particularly difficult pathogen. Also of note is the statistically significant difference between the B hosts and A hosts with respect to no-growth cultures. B hosts had statistically significantly more no-growth cultures ( p<0.03 ). Additional bacterial DNA testing is now standard protocol when no growth occurs in the first 3 to 5 d to further quantify an identifiable organism. These may eventually be identified as *Staphylococcus aureus*, but to date there have been no publications on the most likely organism that does not grow on a culture in orthopedic infections.

The literature on immunological abnormalities is increasing with the advancement in gene identification technologies, as gene defects are being discovered in all major groups of primary immunodeficiencies (Al-Herz et al., 2014; Gallo et al., 2016). Incidence of PID in the literature varies from 1:500 to 1:100000 (Boyle and Buckley, 2007; Hayakawa et al., 1981; Marschall et al., 2015). PID is often underreported, as the diagnosis either never occurs or is substantially delayed (Gallo et al., 2016; Seymour et al., 2005). The orthopedic community needs to consider immunological deficiencies as possible causes of infectious complications related to orthopedic procedures. Retrospective studies of healthcare expenditures, conducted on very large populations in certain geographic areas, reveal that chronic infections are among the highest-cost group (Wodchis et al., 2016). While the cost of the laboratory tests is several hundred (USD 600) dollars, the cost of orthopedic infection is measured in tens of thousands of dollars (USD 30 000–50 000) (Urban, 2006; Fry, 2002). This paper begins to shed light on these contributing factors to infection risk, especially when all other factors are accounted for and managed.

This study has many limitations. First, this a retrospective review documenting laboratory values during an active orthopedic infection. PID diagnosis in our population was suspected based upon the immunologic profile obtained when these patients were actively infected. The comparisons and subgroup comparisons prepared for this paper should not be interpreted as clear evidence of difference but rather allow for exploration and provide a basis for future research. The research staff dedicated to the collection of these data for this paper was not blinded in any manner, creating a bias risk given the purpose of the study. Multiple staff were employed to perform quality control of the dataset and to ensure accuracy. Despite these limitations, this cohort could provide the foundation for future immunological research in the infected orthopedic population. This study, however, does support the importance of immunological workup in the infected traditional A host.

There are many questions raised after documenting these abnormalities. Do inherently lower complement levels predispose the B hosts to more unusual or difficult infections? Do these difficult infections lower the complement levels more significantly in B hosts, making them the “worse host”? The readily identifiable uninfected B hosts would need complement levels to confirm some of these hypotheses. This is currently being investigated and will be reported in a subsequent paper. Further investigation and controlled level-1 studies are needed to elucidate how the host immune system is affected by these variables. Also, a repeat immunology panel after infection eradication is valuable in documenting whether these are persistent immune deficiencies. IgG, IgA, IgM, IgE, and complement lab values were reordered. This has become part of our standard protocol and will be reported in a subsequent paper.

PID should be suspected in infected orthopedic patients who are considered A hosts by the Cierny–Mader classification. Other preoperative warning signs in the orthopedic population include antibiotic treatment with minimal effect, recurrent pneumonia, recurrent deep skin or organ abscesses, fungal infection, chronic sinusitis, and/or need for intravenous antibiotics for infection treatment. We propose adding the immunology workup to the Cierny–Mader classification to correctly quantify these compromised hosts as well as supplement these deficient hosts with IgG when necessary to help combat their active infection.

Host optimization is crucial to ensure proper eradication of infection. Patients with abnormal immunological values would benefit most from a referral to a specialist to monitor immune status and replenish IgG if indicated. For our patients who were diagnosed with primary immunodeficiencies, we developed certain recommendations. These included referral to an immunologist or a hematologist, boosting nutrition status by vitamins and protein supplementation, and additional prophylactic antibiotic use following any subsequent clean surgical procedures.

## 5 Conclusions

This paper highlights the importance of serologic evaluation as an addition to the current Cierny–Mader classification system, which is based on the history and physical for quantifying infection risk. Serologic evaluation in this population meaningfully contributes to the quantification of the patient's “true” host status. IgG supplementation should be considered in all patients with deficiencies as well as patients with recurrent infections. Immunologically deficient A hosts should be quantified as B hosts. IgG deficiencies may be addressed when deemed appropriate by the consulting hematologist/immunologist. Patients with recurrent infections had significantly lower IgM levels than their nonrecurrent infection counterparts.

Future directions will be to evaluate and stratify a large cohort of patients without infection before surgery for immune risk and use preoperative interventions to optimize their outcomes. Based upon these results, we recommend a serologic profile in addition to the history and physical to accurately stratify patients into their proper host status according to the Cierny–Mader classification of orthopedic infection.

## Data Availability

These data are not currently shared in a repository; please direct any requests concerning data to the corresponding author.
